# Wasted Bread as Substrate for the Cultivation of Starters for the Food Industry

**DOI:** 10.3389/fmicb.2020.00293

**Published:** 2020-02-28

**Authors:** Michela Verni, Andrea Minisci, Sonia Convertino, Luana Nionelli, Carlo G. Rizzello

**Affiliations:** ^1^Department of Soil, Plant and Food Sciences, University of Bari Aldo Moro, Bari, Italy; ^2^Valle Fiorita Catering S.r.l, Ostuni, Italy

**Keywords:** bread waste, growth medium, starters, lactic acid bacteria, yeasts, fungi

## Abstract

The amount of bread wasted daily worldwide, throughout its entire lifecycle, from production to distribution, is estimated to be hundreds of tons, therefore representing both economic and environmental issues. This work aimed at the valorization of wasted bread, setting-up a protocol for obtaining a growth medium to be used for the cultivation of food industry microbial starters. The optimization of the protocol included the set-up of parameters for the hydrolysis of the bread nutrient compounds with proteolytic and amylolytic enzymes and the supplementation with nitrogen and/or carbon sources. The suitability of the optimized medium for the growth of lactic acid bacteria, yeasts and fungi from dairy, bakery, and wine industries was assessed. Lactic acid bacteria growth was strongly affected by the quantity and quality of nitrogen sources employed, while yeasts and fungi growth exceeded that obtained with the reference media commonly employed for their cultivation. Wasted bread medium (WBM) represents a realistic option for the valorization and re-use of bread waste, responding to the modern vision of circular economy.

## Introduction

Breadmaking involves industrial and artisanal bakeries all over the world, that together represent one of the largest sectors of the global food industry. Bread production is estimated to be about 100 million tons per year, 65% of which is consumed in Europe (Melikoglu and Webb, 2013). During storage, a complex physical-chemical process defined as staling occurs in bread, mainly driven by the loss of moisture and retrogradation of starch (Narvhus and Sørhaug, 2006). Moreover, bread composition makes it susceptible for microbial attack, which is why preservatives, inhibiting spore, molds and/or yeasts growth, are used to reduce spoilage and ensure safety (Suhr and Nielsen, 2004). Because of the susceptibility to staling and spoiling, a huge amount of bread is discarded by consumers or unsold from the retails causing relevant economic losses. Determining the precise amount of bread wasted during its life cycle is difficult, but it is estimated that hundreds of tons are wasted daily worldwide (Melikoglu and Webb, 2013). Nevertheless, the limited shelf-life is only one of the reasons why a large amount of bread is wasted. Edible bread is wasted also during the manufacturing process, due to substandard products, processing factors, and sometimes to consumer requests. For example, crusts and external layer is removed from loaves in sandwich bread production (up to 40% of the products).

Almost all the not consumed bread is disposed as waste, while a little part is re-used as feed. Nevertheless, over the last decade, many researchers attempted to find recycle alternatives: wasted bread was proposed as substrate to produce, through microbial fermentation, chemicals for pharmaceutical or food industries (Oda et al., 1997; Leung et al., 2012), biofuels (Doi et al., 2009; Pietrzak and Kawa-Rygielska, 2014), or enzymes (Asghar et al., 2002; Cerda et al., 2016). All the proposed strategies can potentially reduce the organic matter disposal into the environment, but none of these completely repay the huge economical loss. For this reason, other alternative possibilities are under investigation: recently, the potential to produce ingredients for food making (Samray et al., 2019), such as the feasibility of bread waste to be used as substrate for **Saccharomyces cerevisiae**biomass production (Benabda et al., 2018), were investigated.

Bread waste contains high concentration of starch (more than 70% on dry matter) and proteins (up to 14% on dry matter) (Melikoglu and Webb, 2013) and treatment with amylases, amyloglucosidases and proteases easily lead to the release of compounds available for the microbial growth (Simpson et al., 2012).

The concept of fermentation, one of the oldest processing technologies, has changed considerably since its dawning. First discovered as efficient tool to increase shelf life of foods, it is also responsible for sensorial, textural and nutritional changes of the fermented ingredient. Its importance in modern-day life is indeed underlined by the wide range of fermented foods on the market and their major impact on nutritional habits (Holzapfel, 2002; Giraffa, 2004). Originally, the process occurred spontaneously due to the development of the microbiota naturally present in the raw material. Nowadays, the necessity to (i) steer the fermentation toward specific technological goals, (ii) avoid failures in the process due to undesirable metabolic activities, and (iii) ensure stability of large-scale productions, enabled the widespread use of selected starter cultures (Holzapfel, 2002; Leroy and De Vuyst, 2004). Fermentation microorganisms include LAB, yeasts and molds. LAB have a long and safe history of application in the production of fermented foods and beverages and their contribution to the processes is substantial. They cause rapid acidification of the raw material through the production of organic acids, aroma compounds, bacteriocins, exopolysaccharides, and enzymes of great importance (Leroy and De Vuyst, 2004). Notwithstanding LAB are the main responsible for dairy, meat, vegetables, and sourdough fermentation (Giraffa, 2004), yeasts can be considered the most important microorganisms in food biotechnology worldwide, exceeding in both production and economic revenues (Kurtzman et al., 2011). The annual world production of *S. cerevisiae* is more than 1 million tons, more than any other industrial microorganism combined (Hansen, 2004; Verstrepen et al., 2006) and the economic values of fermented beverages and foods involving yeasts as starter are enormous: beer, wine and bakery markets alone are worth more than 500 billion dollars (Kurtzman et al., 2011).

When producing commercial starter culture, the choice of the growth media revolves around costs and efficiency of cell production. Competitive growth media should include inexpensive ingredients and enable starters to reach maximum cell density at the lowest time (Heenan et al., 2002). Specific formulations for industrial growth media are rarely reported to maintain the commercial advantage of the company selling the strains. However, low-cost materials include side streams from agricultural and food processes such as molasses, corn syrup or by-products from cheese manufacture (Heenan et al., 2002; Durso and Hutkins, 2003; Kurtzman et al., 2011). Therefore, wasted bread could be considered one of them. The loss of bread occurs throughout the whole supply chain, from production to consumer level, passing through the distribution and this loss results in a major economic impact that cannot be even precisely estimated. Preventing this phenomenon, by putting in place pre-ordering systems to avoid surplus or rethinking sizes and packaging solutions, is one of the options, yet it is necessary to outsmart the process.

In this work, aiming at the valorization and recycle of bread waste, a protocol for obtaining a growth medium to be used for the cultivation of starters was set-up. The protocol for the wasted bread medium (WBM) production was optimized through the use of different commercial enzymes, supplements and bread hydrolysis conditions. The suitability of the medium for the production of lactic acid bacteria (LAB) and yeasts biomasses was investigated.

## Materials and Methods

### Substrate, Enzymes, and Supplements

The substrate used to produce the growth medium was kindly provided by Valle Fiorita Ltd. (Ostuni, Italy) and consisted of white bread cuttings, wasted from sandwiches production. Protein (total nitrogen × 5.7), lipids, ash and moisture contents were determined according to the AACC approved methods 46-11A, 30-10.01, 08-01, and 44-15A, respectively (American Association of Cereal Chemistry [AACC], 2010). Total carbohydrates were calculated as the difference [100- (proteins + lipids + ash + total dietary fibers)]. The determination of total dietary fibers was carried out by AOAC approved method (Horwitz and Latimer, 2006). Proteins, lipids, carbohydrates and ash were expressed as % of dry matter (d.m.).

Commercial amylolytic and proteolytic enzyme preparations were used in this study. Novamyl, a maltogenic amylase (1500 U/g) from *Bacillus subtilis* (Novozyme, Denmark) and amyloglucosidase (6000 U/g) from *Aspergillus niger* (Sigma-Aldrich) were used to hydrolyze starch. Proteolytic enzymes were Veron PS (227 hemoglobin units on the tyrosine basis/g) obtained from *Aspergillus oryzae* (AB Enzymes GmbH, Darmstadt, Germany) and Neutrase, an endo-protease (1.5 AU/g) from *Bacillus amyloliquefaciens* (Novozyme, Denmark). Fungal proteases from *Aspergillus oryzae* (E1; 500,000 hemoglobin units on the tyrosine basis/g) and *A. niger* (E2; 3000 spectrophotometric acid protease units/g) were purchased from BIOCAT Inc. (Troy, VA, United States). Except for the amyloglucosidase (provided by Sigma-Aldrich for analytical use), all the enzymes are routinely used as improvers in bakery industry.

### Microorganisms

*Lactobacillus plantarum* LB1 and *Saccharomyces cerevisiae* E10, respectively isolated from wheat germ (Rizzello et al., 2010) and wheat flour sourdough, and belonging to the Culture Collection of the Department of Soil, Plant and Food Sciences (University of Bari, Italy), were chosen as indicator microorganisms for the set-up and the optimization of the protocol for WBM production.

*Lactobacillus plantarum* LB1 and *S. cerevisiae* E10 were routinely propagated on de man, rogosa and sharpe (MRS) (Oxoid, Basingstoke, Hampshire, United Kingdom) at 30°C and on Sabouraud Liquid Medium (Oxoid) at 25°C, respectively. LAB and yeast strains were cultivated until the late exponential phase of growth was reached (ca. 10 h for LAB and 20 h for yeast), harvested by centrifugation at 9000 × *g* for 10 min at 4°C, washed twice in 50 mM sterile phosphate buffer (4°C, pH 7.0), resuspended in sterile distilled water and used to inoculate WBM (4% [v/v], corresponding to initial cell density of ca.7.0 log cfu/g).

The growth of the strains in WBM was estimated by measuring the optical density (OD) at 620 nm using a spectrophotometer model Ultrospec 3000 (Pharmacia Biotech, Sweden), with cuvettes with a 1-cm light path). Once the medium formulation and protocol was set-up, the changes in turbidity recorded with the OD, were confirmed by plating the cultures on MRS and Sabouraud agar.

To assess the suitability of the WBM as substrate for LAB cultivation, other strains were included in the study. *Lb. plantarum* C2, C48, H48, T6B4, T0A10, T6C16, 18S9, MRS1, 1A7, PU1, PRO17, *Lactobacillus rossiae* LB5, T0A16, *Lactobacillus brevis* H46, MRS4, AM7, *Pediococcus pentosaceus* H11, T1A13, I76, I214, I02, I014, F01, OA1, S3N3, BAR4, *Pediococcus acidilactici* 10MM0, *Pediococcus* sp. I56, *Leuconostoc mesenteroides* 12MM1, I57, *Weissella confusa* KAS3, and NEY6, all belonging to the Culture Collection of the Department of Soil, Plant and Food Sciences (University of Bari, Italy) were used. Before inoculation in WBM, LAB strains were cultivated as described above.

### Kinetics of Growth

Microbial kinetics of growth were determined and modeled in agreement with the Gompertz equation, as modified by Zwietering et al. (1990): *y* = *k* + *A exp{−exp[(*μ*max e/A)(*λ*−t)* + *1]}*; where *y* is the OD_620_; *k* is the initial level of the dependent variable to be modeled (OD_620_); *A* is the cell density variation (between inoculation and the stationary phase); μ*max* is the maximum growth rate expressed as OD_620_ units/h; λ is the length of the lag phase measured in hours. The experimental data were modeled by the non-linear regression procedure of the Statistica 8.0 software (Statsoft, Tulsa, United States).

### Set-Up of the Protocol for WBM Production

Bread waste was finely grinded by the laboratory mill Ika-Werke M20 (GMBH, and Co. KG, Staufen, Germany) and added to distilled water in a percentage ranging from 10 to 50%.

Proteolytic and amylolytic enzymes, singly or combined, were then added. Novamyl and Neutrase (Novozyme) were each used at 0.5 g/l, amyloglucosidase (Sigma-Aldrich) was added at 0.1 g/l, Veron PS (AB Enzyme) was employed at the concentration of 25 mg/100 kg of proteins, whereas 200 ppm of both the fungal proteases E1 and E2 were added at the suspensions (Table 1).

**TABLE 1:** Combinations used to set-up and optimize wasted bread medium (WBM) production.

Sample	Bread waste (%)	Enzymes employed	Hydrolysis conditions	Supplements added
M_1_	20	Novamyl (0.5 g/l)	55°C × 24 h	–
M_2_	20	Novamyl (0.5 g/l) – Neutrase (0.5 g/l)	55°C × 24 h	–
M_3_	20	Novamyl (0.5 g/l) – Neutrase (0.5 g/l)	55°C × 24 h	Glucose (20 g/l)
M_4_	20	Novamyl (0.5 g/l) – Neutrase (0.5 g/l)	55°C × 24 h	Glucose (20 g/l) – Yeast extract (5 g/l)
M_5_	20	Novamyl (0.5 g/l) – Neutrase (0.5 g/l)	55°C × 6 h	Glucose (20 g/l) – Yeast extract (5 g/l)
M_6_	20	Novamyl (0.5 g/l) – Neutrase (0.5 g/l)	55°C × 12 h	Glucose (20 g/l) – Yeast extract (5 g/l)
M_7_	20	Novamyl (0.5 g/l) – Neutrase (0.5 g/l)	55°C × 48 h	Glucose (20 g/l) – Yeast extract (5 g/l)
M_8_	20	Novamyl (0.5 g/l), Amyloglucosidase (0.1 g/l) – Neutrase (0.5 g/l)	55°C × 24 h	Yeast extract (5 g/l)
M_9_	20	Novamyl (0.5 g/l), Amyloglucosidase (0.1 g/l) – Veron PS (25 mg/100 kg of proteins)	55°C × 24 h	Yeast extract (5 g/l)
M_10_	20	Amyloglucosidase (0.1 g/l) – Veron PS (25 mg/100 kg of proteins)	55°C × 24 h	Yeast extract (5 g/l)
M_11_	20	Novamyl (0.5 g/l), Amyloglucosidase (0.1 g/l) – Veron PS (5 mg/l)	30°C × 24 h	Yeast extract (5 g/l)
M_12_	20	Amyloglucosidase (0.1 g/l) – Veron PS (5 mg/l)	30°C × 24 h	Yeast extract (5 g/l)
M_13_	20	Novamyl (0.5 g/l) – Veron PS (5 mg/l)	55°C × 24 h	Yeast extract (5 g/l)
M_14_	20	Novamyl (0.5 g/l) – Neutrase (0.5 g/l)	55°C × 24 h	Yeast extract (5 g/l)
M_15_	20	Novamyl (0.5 g/l) – Neutrase (0.5 g/l)	55°C × 24 h	Yeast extract (10 g/l)
M_16_	20	Novamyl (0.5 g/l) – Neutrase (0.5 g/l)	55°C × 24 h	Yeast extract (5 g/l) – Peptone (5 g/l)
M_17_	20	Novamyl (0.5 g/l) – Neutrase (0.5 g/l)	55°C × 24 h	Yeast extract (10 g/l) – Peptone (10 g/l)
M_18_	20	Novamyl (0.5 g/l) – Neutrase (0.5 g/l)	55°C × 24 h	Yeast extract (20 g/l)
M_19_	20	Novamyl (0.5 g/l) – Neutrase (0.5 g/l)	55°C × 24 h	Peptone (20 g/l)
M_20_	20	Novamyl (0.5 g/l) – Neutrase (0.5 g/l)	55°C × 24 h	Tryptone (20 g/l)
M_21_	20	Novamyl (0.5 g/l) – Neutrase (0.5 g/l)	55°C × 24 h	Meat extract (20 g/l)
M_22_	20	Novamyl (0.5 g/l) – Neutrase (0.5 g/l)	55°C × 24 h	WPC (20 g/l)
M_23_	20	Novamyl (0.5 g/l) – Neutrase (0.5 g/l)	55°C × 24 h	WPI (20 g/l)
M_24_	20	Novamyl (0.5 g/l) – Neutrase (0.5 g/l)	55°C × 24 h	Yeast extract (30 g/l)
M_25_	20	Novamyl (0.5 g/l) – Neutrase (0.5 g/l)	55°C × 24 h	Peptone (30 g/l)
M_26_	20	Novamyl (0.5 g/l) – Neutrase (0.5 g/l)	55°C × 24 h	Tryptone (30 g/l)
M_27_	20	Novamyl (0.5 g/l) – Neutrase (0.5 g/l)	55°C × 24 h	Meat extract (30 g/l)
M_28_	20	Novamyl (0.5 g/l) – Neutrase (0.5 g/l)	55°C × 24 h	WPC (30 g/l)
M_29_	20	Novamyl (0.5 g/l) – Neutrase (0.5 g/l)	55°C × 24 h	WPI (30 g/l)
M_30_	20	Novamyl (0.5 g/l) – Neutrase (0.5 g/l)	55°C × 24 h	Yeast extract (10 g/l) – Peptone (10 g/l) – Tryptone (10 g/l)
M_31_	20	Novamyl (0.5 g/l) – Neutrase (0.5 g/l)	55°C × 24 h	Yeast extract (10 g/l) – Peptone (10 g/l) – Meat extract (10 g/l)
M_32_	20	Novamyl (0.5 g/l) – Neutrase (0.5 g/l)	55°C × 24 h	Yeast extract (10 g/l) – Peptone (10 g/l) – WPC (10 g/L)
M_33_	20	Novamyl (0.5 g/l) – Neutrase (0.5 g/l)	55°C × 24 h	Yeast extract (10 g/l) – Peptone (10 g/l) – WPI (10 g/l)
M_34_	10	Novamyl (0.5 g/l) – Neutrase (0.5 g/l)	55°C × 24 h	Yeast extract (30 g/l)
M_35_	10	Novamyl (0.5 g/l) – Neutrase (0.5 g/l)	55°C × 24 h	Peptone (30 g/l)
M_36_	10	Novamyl (0.5 g/l) – Neutrase (0.5 g/l)	55°C × 24 h	Tryptone (30 g/l)
M_37_	10	Novamyl (0.5 g/l) – Neutrase (0.5 g/l)	55°C × 24 h	Meat extract (30 g/l)
M_38_	10	Novamyl (0.5 g/l) – Neutrase (0.5 g/l)	55°C × 24 h	Yeast extract (10 g/l) – Peptone (10 g/l) – WPC (10 g/l)
M_39_	10	Novamyl (0.5 g/l) – Neutrase (0.5 g/l)	55°C × 24 h	Yeast extract (10 g/l) – Peptone (10 g/l) – WPI (10 g/l)
M_40_	30	Novamyl (0.5 g/l) – E1 (200 ppm) – E2 (200 ppm)	37°C × 24 h	–
M_41_	50	Novamyl (0.5 g/l) – E1 (200 ppm) – E2 (200 ppm)	37°C × 24 h	–
M_42_	50	Novamyl (0.5 g/l) – E1 (200 ppm) – E2 (200 ppm)	37°C × 24 h	Yeast extract (5 g/l)
M_43_	50	Novamyl (0.5 g/l) – E1 (200 ppm) – E2 (200 ppm)	37°C × 24 h	Yeast extract (10 g/l)
M_44_	50	Novamyl (0.5 g/l) – E1 (200 ppm) – E2 (200 ppm)	37°C × 24 h	WPC (10 g/l)

Bread suspensions were then incubated at 30, 37, or 55°C for 6, 12, 24, or 48 h depending on the enzymes optimal conditions suggested by the manufacturers. After the incubation, mixtures were centrifugated (14000 rpm for 20 min) to separate not hydrolyzed solid residues and supernatants were collected. Aiming at maximizing microbial growth, the medium was supplemented with glucose [10 or 20 g/l], yeast extract [5, 10, 20, or 30 g/l], peptone [5, 10, 20, or 30 g/l], tryptone [10, 20, or 30 g/l], and meat extract [10, 20, or 30 g/l] (Oxoid). Whey protein isolates (WPI; [10, 20, or 30 g/l]) and concentrate (WPC; [10, 20, or 30 g/l]) (Sigma-Aldrich) were also used. Supplements were added to the medium singly or in mixture as reported in Table 1.

The medium was adjusted to pH 5.6 (using a pH-meter Model 507, Crison, Milan, Italy) and sterilized at 121°C for 15 min. To avoid interferences due to different turbidimetry during the OD measurement, the media were further filtered using sterile membrane size of 0.2 μm cut-off (Millipore, United States).

WBM obtained from the optimized protocol was freeze-dried to assess the suitability of the medium to be rehydrated.

### WBM Chemical Characterization

To establish a correlation between the chemical composition of the media and microbial growth, glucose, peptides and amino acids concentrations were determined for each experimental condition. Glucose content was determined by using the D-Fructose/D-Glucose Assay Kit (Megazyme Int., Ireland) following the manufacturer’s instructions. Peptides concentration was estimated by the o-phtaldialdehyde (OPA) method as described by Church et al. (1983). A standard curve prepared using tryptone (0.25 to 1.5 mg/ml) was used as reference. Total free amino acids (TFAA) concentration was analyzed by a Biochrom 30 series Amino Acid Analyzer (Biochrom Ltd., Cambridge Science Park, United Kingdom) with a Na-cation-exchange column as described by Rizzello et al. (2008).

### Microbial Dry Biomass

Dry biomass was determined for Lb. plantarum LB1 and S. cerevisiae E10 grown in MRS and Sabouraud or WBM. Strains were cultivated until the late exponential phase of growth was reached and harvested by centrifugation. Twenty milliliters of culture were centrifuged (9000 × g) in pre-weighed tubes and then the pellet was dried for 24 h at 60°C. Pellets were equilibrated by exposure to silica salts in a desiccator. Centrifuge tubes were reweighed, and the weight of biomass calculated.

### WBM as Substrate for the Cultivation of Food Industry Starters

Starters routinely employed in bread, dairy, and wine industry were grown in WBM to assess the suitability of the medium to be used as substrate for biomass production. Before inoculation, Lactobacillus delbrueckii subsp. bulgaricus 2B and Oenococcus oeni O1 were propagated in MRS, Streptococcus thermophils in M17 (Oxoid), S. cerevisiae LNN2 and Enolievito (Enol) in Sabouraud at 25°C for 24–48 h, Acetobacter sp. in Acetobacter Broth (Glucose) (Himedia) in aerobic conditions at 30°C for 48 h, whereas Penicillium roqueforti DPPMAF1 was grown in Potato Dextrose Agar (Oxoid) at 25°C for 72 h. Except for S. cerevisiae Enol, which is a commercial strain selected for wine making (Vebi, Italy), all the above species belonged to the Culture Collection of the Department of Soil, Plant, and Food Sciences (Bari, Italy).

### Effect of Preservatives Residues

Aiming at evaluating the effect of chemical preservatives, commonly added to bread to prevent spoilage, on the growth of P. roqueforti, calcium propionate and potassium sorbate (Sigma-Aldrich) were added to WBM (containing 1.5% agar). The inhibitory activity of the preservatives was assayed based on hyphal radial growth rate of fungi. The salt solutions were sterilized by filtration on 0.22 μm membrane filters (Millipore Corporation, Bedford, MA 01730) and added to the WBM at the final concentration of 0.15, 0.1125, 0.075, 0.0375% (wt/vol) for propionate and 0.1, 0.075, 0.05, 0.025% (wt/vol) for sorbate, corresponding to 25, 50, 75 and 100% of the maximum concentration allowed in bread by law (Reg. CE N. 638/2016). After mixing, aliquots of 20 ml were poured into Petri plates (90 mm diameter). Control plates contained WBM supplemented with sterile water. Plates were incubated aerobically at 25°C and the radial growth of mycelia (colony diameter, mm) in all plates was measured 6 days after inoculation. Each datum point is the mean of at least four measurements of a growing colony. The percentage of growth inhibition was calculated from mean values as follows: percentage of inhibition = [(mycelial growth under control conditions - mycelial growth in the presence of the preservatives)/mycelial growth under control conditions] × 100.

### WBM as Substrate for the Enumeration of Cultivable Bacteria and Yeasts

WBM was used for the plate count of LAB and yeasts in food matrices (wheat flour sourdough, yogurt, and grape must) and the results were compared to those obtained with the laboratory media usually employed in LAB and yeasts count (MRS and Sabouraud agar, respectively).

In particular, freeze-dried WBM (14.5%) was supplemented with agar (1.5%) and sterilized at 121°C for 30 min. For the analysis, 10 g of each food sample were homogenized with 90 ml of sterile peptone water (0.1% [wt/vol] peptone, 0.85% [wt/vol] NaCl) solution. Lactic acid bacteria were counted at 30°C for 48 h under anaerobiosis using both WBM and MRS agar medium supplemented with cycloheximide (0.1 g/l). The number of yeasts was estimated at 30°C for 48 h on WBM and Sabouraud dextrose agar (SDA) supplemented with chloramphenicol (0.1 g/l).

### Statistical Analysis

Data were subjected to one-way ANOVA; pair-comparison of treatment means was obtained by Tukey’s procedure at P < 0.05, using the statistical software Statistica 8.0 (StatSoft Inc., Tulsa, United States). Modeling aimed at describing LAB and yeasts growth rate as function of the independent variables (glucose, peptides and amino acids concentrations). A software package (Statistica for Windows) was used to fit the second-order model to the independent variables using the following equation:

$$\gamma =\sum B_{i}\,\chi_{i}+\sum B_{\mathrm{ii}}\,\chi_{i}^{2}+\sum B_{\mathrm{ij}}\,\chi_{i}\,\chi_{j}$$

where γ is the dependent variable (growth rate) to be modeled, B_*i*_, B_*ii*_ and B_*ij*_ are regression coefficients of the model, and χ_*i*_ and χ_*j*_ are the independent variables in coded values. This model allowed the evaluation of the effects of linear, quadratic and interactive terms of the independent variable on the dependent variables. Three-dimensional surface plots were drawn to illustrate the main and interactive effects of the independent variables on the dependent ones.

## Results

### Set-Up and Optimization of the Protocol for WBM Production

The bread employed as substrate ingredient had a moisture of 35.7% and its chemical composition, was the following: proteins, 11.40 ± 0.49% (d.m.); fats, 6.25 ± 0.21% (d.m.); carbohydrates, 77.44 ± 3.11% (d.m.); dietary fibers, 2.35 ± 0.15% (d.m.).

**FIGURE 1:**
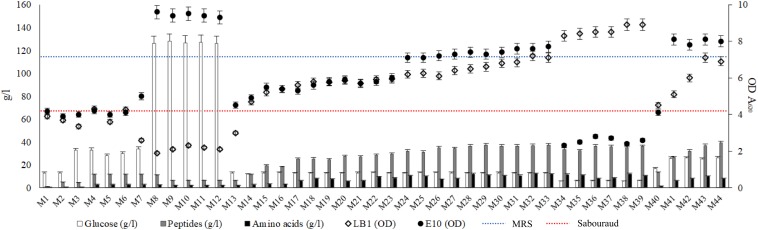
Concentration of glucose, peptides and amino acids (g/l) of the media obtained from wasted bread **(Left axis)**. Dots represent the distribution of the optical density (A_620 nm_) values of *Lactobacillus plantarum* LB1 and *Saccharomyces cerevisiae* E10 after 24 h of incubation at 30°C in the experimental media **(Right axis)**. Dotted lines indicate the growth of the same strains in reference media, MRS (blue) and Sabouraud (red) for LAB and yeasts, respectively. Experimental media (M_1_ to M_44_) correspondence is reported in Table 1.

Aiming at setting up the protocol for the production of a growth medium obtained from wasted bread, more than 40 different production options were tested (Table 1). The following parameters were changed: amount of bread employed, enzyme type and concentration, time and temperatures of incubation, type and concentration of supplement added. The cell density of *Lb. plantarum* LB1 and *S. cerevisiae* E10, inoculated as indicator microorganisms, was measured after incubation for all the WBM samples obtained by varying the production protocols parameters, and compared to that obtained with media commonly used to propagate LAB and yeasts (Figure 1). To clarify the effect of the media chemical composition on LAB and yeasts growth, each substrate was characterized for the content of glucose, peptides and free amino acids (Figure 1).

M_1_, obtained hydrolyzing bread only with the Novamyl amylase, had a glucose content of 13.02 ± 0.09 g/l and less than 1 g/l of peptides and amino acids, whereas the addition of the proteolytic enzyme Neutrase (M_2_) allowed the release of peptides and amino acids up to 4.7 and 0.5 g/l, respectively. Although the composition of both these media enabled a relevant growth for *S. cerevisiae* E10, that of *Lb. plantarum* LB1 was markedly lower than in MRS (Figure 1). M_2_ was therefore supplemented with yeast extract and/or glucose (M_4_ and M_3_). Hydrolysis incubation time was also modified, detecting slight but significant (*P* < 0.05) changes in glucose concentration. Compared to M_4_, the shortening of the incubation time to 6 or 12 h (M_5_ or M_6_) and the extension to 48 h (M_7_) respectively caused the decrease (34 and 12%) or the increase (6%) of the amount of glucose released.

None of the above described conditions enabled a growth, for *Lb. plantarum* LB1, comparable to that of the reference medium, therefore other enzymes were tested. The use of amyloglucosidase (M_8_−M_12_) caused the release of glucose up to 126.24 ± 0.41 g/l. To improve the proteolysis and, as consequence, the availability of easily usable nitrogen compounds, Veron PS instead of Neutrase was used (M_9_**_–_**_13_). Incubation temperature was also changed to 30°C, the optimal value for Veron PS activity. Despite yeasts growth was the highest in these conditions (M_8_**_–_**_12_), that of LAB worsened. In MRS and Sabouraud glucose concentration was 20.56 ± 0.14 and 18.64 ± 0.46 g/l, respectively, whereas peptides and amino acids were 24.85 ± 0.23 and 4.24 ± 0.05 g/l in MRS and 8,89 ± 0,16 and 1,97 ± 0,07 g/l for Sabouraud. Therefore, the content in peptides and amino acids was identified as potential limiting factor of the bacterial growth. The use of protein supplements was therefore investigated.

The addition of yeast extract alone (M_15_) or with peptone (M_16_), for a total of 10 g/l, slightly improved *Lb. plantarum* LB1 growth. Several formulations containing peptone, tryptone, yeast extract, WPC and WPI, up to 20 g/l (M_17_**_–_**_23_), increased peptides and amino acids content, respectively ranging from 24.51 ± 0.29 to 29.32 ± 0.33 g/l and from 5.65 ± 0.18 to 8.75 ± 0.23 g/l. Consequentially, bacterial growth increased compared to previous media, yet still lower than MRS. The addition of peptone, tryptone, yeast extract, WPC and WPI, alone or in combination, allowed to obtain peptide concentrations up to 40 g/l (M_24_**_–_**_39_), whereas amino acids concentration reached 10 g/l. On the basis of the demonstrated possibility to release high amounts of glucose from bread, media from M_34_ to M_39_ were obtained using only 10% of bread and by adding 30 g/l of supplements. In this condition, the content of glucose after hydrolysis decreased to ca. 6 g/l, and *S. cerevisiae* growth was affected dropping below the reference medium (Figure 1), while the highest LAB growth was observed when yeast extract, peptone and WPI were used together (M_33_−M_39_).

Despite the good results observed for LAB and considering that the main substrate for the medium was supposed to be wasted bread, the formulation was further reconsidered. Instead of focusing on the protein sources added, proteolysis efficiency was improved. The proteolytic enzymes E1 and E2 allowed the efficient release of peptides and amino acids from proteins, therefore the concentration of supplements was decreased. The bread percentage in medium formulation was therefore increased to guarantee a sufficient organic nitrogen supply (M_40_**_–_**_44_) in presence of low amount or in absence of supplements. The temperature of incubation was lowered to 37°C, optimal for E1/E2 activity. Compared to M_2_ (Neutrase addition), peptides and amino acids released during the hydrolysis were, in these conditions, ca. 2- and 4-fold higher, respectively. Media obtained with the formulation including 50% of bread, containing up to 25 g/l of glucose, promoted yeasts growth, yet only the addition of 10 g/l of yeast extract (M_43_) allowed a bacterial growth comparable to that of MRS.

Overall, final LAB cell density was lower in almost all the WBM formulations tested (M_1__–__30_ and M_40__–__42_), compared to MRS (Figure 1). Few conditions, corresponding to those having the highest organic nitrogen supplementation (from M_30_ to M_39_ and M_43_−M_44_), matched or exceeded the OD values obtained with MRS. On the contrary, except for M_34__–__39_, *S. cerevisiae* growth resulted in almost all the conditions higher than that observed in the reference media, especially in thesis characterized by the highest glucose concentration (M_8__–__12_).

**FIGURE 2:**
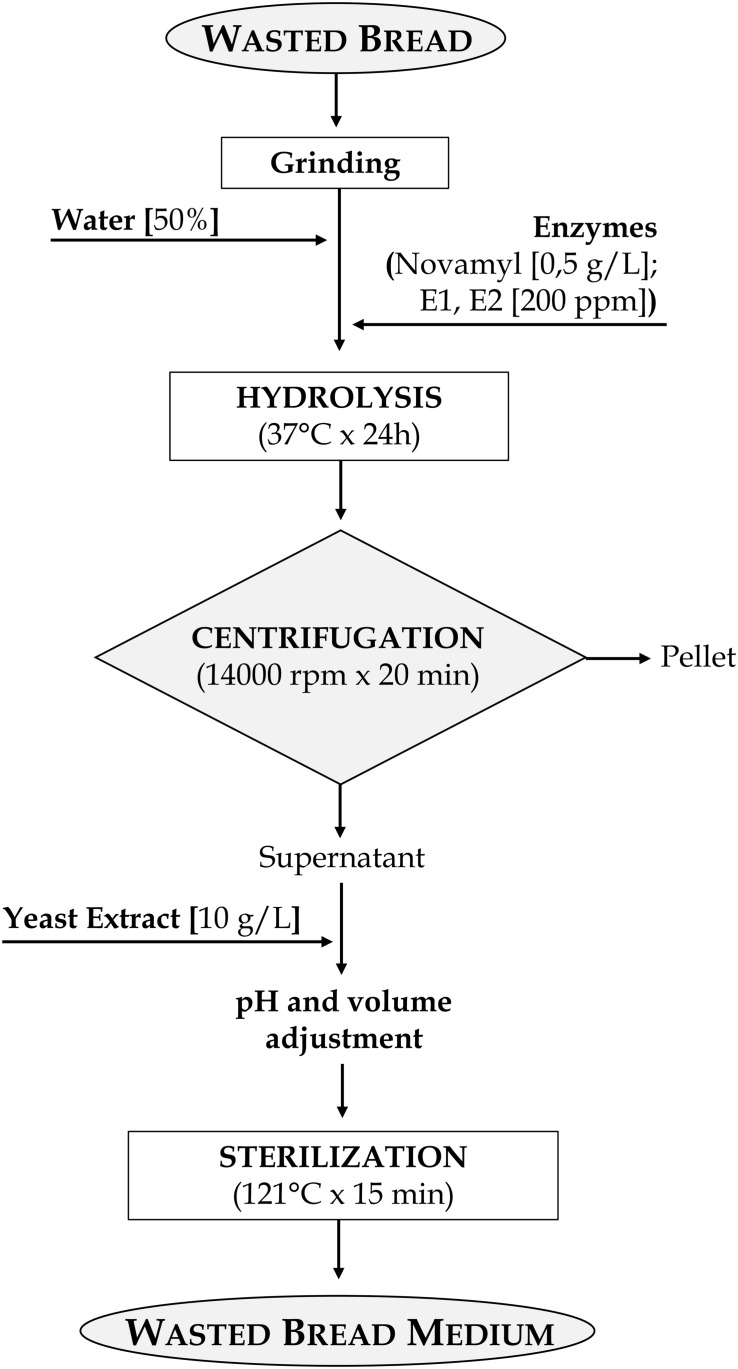
Flowchart of the protocol for the production of wasted bread medium (WBM).

Notwithstanding M_34__–__39_ had the best performances for LAB in terms of final cell density, M_43_ was selected for WBM production since corresponding to the best growth performances in absence of protein supplements. Therefore, it was used in further analysis. WBM, obtained according to the detailed protocol reported in Figure 2, after filtration, contained 24.53 ± 0.29, 37.08 ± 0.39, and 10.76 ± 0.12 g/l of glucose, peptides and amino acids, respectively. The protocol for the WBM production was deposited with the number 102019000017408 at the Italian Patent and Trademark Office on the 27th of September 2019.

The dry biomass of *Lb. plantarum* LB1 and *S. cerevisiae* E10 grown in WBM was determined. No statistical differences (*P* > 0.05) were found between the bacteria strain grown in MRS and WBM (2.45 ± 0.09 and 2.27 ± 0.12 mg/ml, respectively). On the contrary, the biomass of the yeast was markedly and significantly higher in WBM (8.58 ± 0.25 mg/ml) compared to that obtained in Sabouraud (6.39 ± 0.11 mg/ml). Aliquots of the WBM produced with the selected protocol were freeze-dried, with a yield of 145 ± 3 g of powdered medium for liter.

### WBM for LAB and Yeast Enumeration

Agar containing-WBM was used to enumerate cultivable bacteria and yeasts in food matrices, and the results compared with those obtained using the conventional laboratory media. The estimated cell density of presumptive lactic acid bacteria in the yogurt sample was 8.08 ± 0.12 and 8.26 ± 0.10 log cfu/g, when WBM and MRS were used, respectively. When wheat sourdough was analyzed, 9.26 ± 0.08 and 9.44 ± 0.07 log cfu/g of LAB were respectively enumerated with WBM and MRS. In both the cases, no significant differences (*P* > 0.05) were found.

For yeasts, also enumerated in wheat sourdough and grape must samples, no significant (*P* > 0.05) differences were found by comparing the results obtained using WBM or Sabouraud agar media. In particular, cell densities of 7.46 ± 0.11 and 4.81 ± 0.09 log cfu/g were respectively found in wheat sourdough and grape must.

### WBM for LAB Cultivation

**FIGURE 3:**
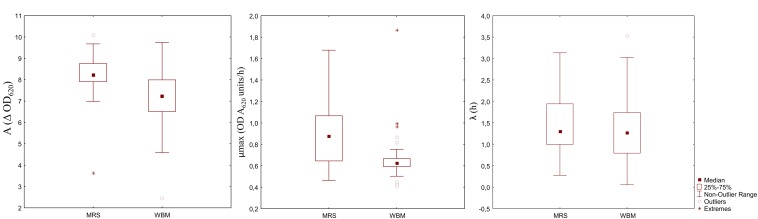
Box-plot showing the distribution of 33 LAB strains based on the parameters of the kinetics of growth: cell density variation between inoculation and the stationary phase **(A)**, maximum growth rate **(B)**, and length of the lag phase **(C)**.

Aiming at evaluating the suitability of WBM to the cultivation of different LAB species and strains, the media acidification and the growth of 33 different starter LAB were monitored. After 24 h of incubation, the pH of WBM ranged from 3.65 to 4.09, while MRS final pH values were slightly higher (3.94 – 4.50). Except for 11 strains (*Pediococcus* spp. I56, *P. pentosaceus* T1A13, BAR4, S3N3, *Lb. plantarum* C48, T6C16, T0A10, PU1, PRO17, *Lb. rossiae* LB5 and *W. confusa* KAS 3), all LAB had a latency phase similar or slightly but significantly lower than the respective strain growth in MRS (Figure 3). Overall, the maximum velocity of growth was significantly lower in WBM, compared to MRS. Only *P. pentosaceus* BAR4, T1A13, I02, I014, I76, I214, F01 and *Leuc. mesenteroides* I57 showed higher μ*max* (*P* < 0.05) when cultivated in WBM. Whereas, the cell density variation between the inoculum and the stationary phase was significantly higher in WBM only for *Lb. plantarum* 18S9, C2 and *Lb. brevis* H46. *Lb. plantarum* 1A7, *P. pentosaceus* S3N3 and *P. acidilactici* 10MM0, were the strains that worse adapted to the medium.

### WBM for Cultivation of Starters Selected for Dairy and Enological Uses

The medium obtained from wasted bread was tested as substrate for the cultivation of other food industry starters. Two LAB species commonly employed in dairy industry, *Lb. delbrueckii* subsp. *bulgaricus* and *St. thermophilus* were included in the evaluation. Compared to MRS, *St. thermophilus* growth was slightly higher in WBM whereas an opposite trend was observed for *Lb. delbrueckii* subsp. *bulgaricus*. Nevertheless, in both cases no statistical differences were found between the two media. On the contrary, when the medium formulation was modified using 10g/l of WPC instead of yeast extract (M_44_), both starters growth was boosted. *P. roqueforti*, was also propagated on WBM and a growth 42% higher than PDA was reported.

Among the wine industry starters, one more LAB and one yeast, commonly employed for malolactic and alcoholic fermentation respectively, were also tested. Compared to the reference media, when cultivated in WBM both starters, *O. oeni* O1 and *S. cerevisiae* Enol, overgrew reaching a final density almost 0.3 and 1.0 log cycle higher than the control, respectively.

### Evaluation of the Bread Preservatives Concentration on *P. roqueforti* Growth

**FIGURE 4:**
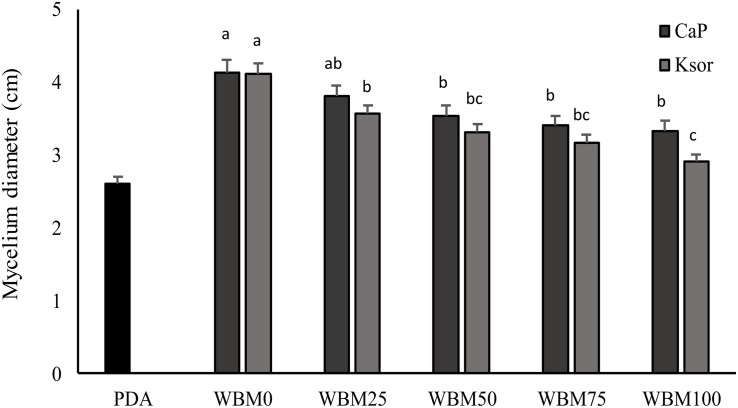
Effect of chemical preservatives, calcium propionate (CaP) and potassium sorbate (Ksor), on the growth of *Penicillium roqueforti* DPPMAF1. Concentrations corresponding to the 25, 50, 75, and 100% of that maximum allowed by the current legislation for baked goods were added to the wasted bread medium (WBM). PDA was used as control medium. Data are the means of three independent analyses. Error bars indicating the standard deviation are represented. ^a–c^Values with different superscript letters differ significantly (*P* < 0.05).

The addition of food preservatives to WBM slightly influenced *P. roqueforti* growth, which proportionally decreased at higher concentrations of calcium propionate and potassium sorbate (Figure 4). A reduction in the mycelium growth varying from 8 to 19% was observed when calcium propionate was added. Whereas, higher inhibition (from 13 to 29%) was found for potassium sorbate. Nevertheless, even when preservatives concentration was the highest, fungal growth in WBM was significantly (*P* < 0.05) higher than PDA.

## Discussion

The use of selected microorganisms as viable starter cultures is largely adopted in the modern food industry to address the fermentation processes toward specific sensory, nutritional and functional targets, to extend the shelf-life, and to ensure the standardization of the product quality (Leroy and De Vuyst, 2004). Accordingly, suitable and cheap substrates to produce large biomasses of starters are required by the industry.

This study concerned the set-up of a growth medium for the cultivation of food industry starter using wasted bread as the main substrate. Due to the great relevance of LAB and yeasts in food industry, one strain of each, both isolated from sourdough environment were chosen as indicators during the optimization of the production protocol.

**FIGURE 5:**
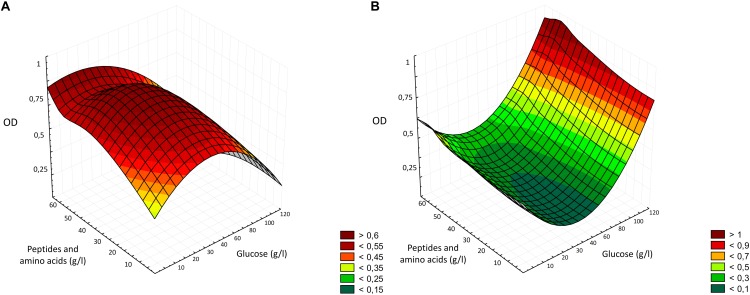
Three-dimensional surface plot of the interaction of glucose and peptides and amino acids concentration (g/l) on the growth of *Lactobacillus plantarum* LB1 **(A)** and *Saccharomyces cerevisiae* E10 **(B)**.

The optimization of the medium followed the classical approach of the one-factor-at-a-time (OFAT) experiments, which considered the variation of one factor at a time (Singh et al., 2017), removing, supplementing or replacing carbon and nitrogen sources, whether they were actual supplements or enzymes or conditions able to modify their content. The two major components of bread are starch and proteins, therefore, the first two enzyme employed were Novamyl and Neutrase, commonly used in bakeries. Novamyl is a maltogenic amylase, that modifies starch delaying its retrogradation, whereas Neutrase is an endopeptidase capable of hydrolyzing gluten network, improving the workability of doughs (Novozymes, 2018). Since carbon is the most important component in a medium, acting as energy source and playing an important role in the growth, often influencing the biomass formation (Singh et al., 2017), the following steps included the addition of glucose as supplement or the use of amyloglucosidases, which has glucose as end-product. Aiming at selecting the optimal production protocol and WBM formulation, all the data resulting from the media chemical characterization and LAB and yeasts growth were factored into three-dimensional surface plots (Figure 5). As showed by the statistical elaboration, a deleterious effect on LAB growth was observed at higher concentration of glucose, probably due to the osmotic stress (Figure 1). When the osmolality of the environment changes, in order to survive and prevent damages to essential cell functions, bacteria adapt by accumulating compatible solutes. However, *Lb. plantarum* and other LAB have limited or no possibilities to synthesize compatible solutes (van de Guchte et al., 2002). Although, it was reported that the optimal condition for the growth of lactobacilli includes concentration of glucose of 4% (Gomes and Malcata, 1999), the media most employed for LAB cultivation contain no more than 2% of glucose, confirming the results obtained during the optimization process (Figure 1). Moreover, after the consumption of carbohydrates, accumulation of lactic acid occurs, resulting in a pH decrease, and when pH 3.6–4.0 is reached, growth ceases for many *Lactobacillus* species (De Vos et al., 2011). Therefore, it is possible that both acid and osmotic stresses contributed to a lower growth of *Lb. plantarum* in the media with higher glucose concentrations. However, the average final pH observed in WBM was only 0.27 lower than that of MRS. Yeasts, on the contrary, can often grow on media with concentrations of sugar that are high enough to inhibit the development of many bacteria and are usually resistant to high osmotic pressure, tolerating low water activity, explaining why, in the interval considered, the higher was glucose concentration, the higher yeasts growth occurred (Figure 5). Many species can indeed grow well in glucose concentrations up to 40% (Kurtzman et al., 2011). Therefore, despite the main enzymatic activity of the commercial preparation Novamyl enables the formation of maltose residues from starch, the glucose liberated (ca. 55 mg/g of bread) was enough for microbial growth, making useless any further addition. Like carbon, the selection of nitrogen source and its concentration in the media also play a crucial role in microbial metabolisms (Singh et al., 2017), which is why, a great deal of effort was put to find the best combination enzyme/substrates supporting microbial growth. Hence, the change of the protease and the addition of different concentration of yeast extract, peptone, tryptone, meat extract, WPI and WPC, were further evaluated. While yeasts can utilize a wide variety of nitrogen sources, both organic and inorganic (Kurtzman et al., 2011), LAB have more complex requirement, needing both proteins and their breakdown products. For example, the presence of thiol groups contained in whey protein favors the growth of some lactobacilli, whereas peptone stimulates acid production (Gomes and Malcata, 1999), and some LAB species also require specific amino acids (Durso and Hutkins, 2003). As a matter of fact, the media containing WPI and WPC, especially in combination with peptone and yeast extract (M_32__–__33_, M_38__–__39_) effectively promoted LAB growth (Figure 1), exceeding MRS performances. Peptone and yeast extract at concentration of 20 g/l have been reported to be among the optimal conditions for LAB growth and represent the main constituent of the media for LAB (Gomes and Malcata, 1999; Schillinger and Holzapfel, 2003).

Nevertheless, these formulations contained a low amount (10 or 20%) of wasted bread and a relevant addition of supplements was required. The expenses related to the supplements and the enzymes, for a specific amount of medium produced, reached those of MRS, making not worth the effort. Being the costs the factor with the highest weight in the choice of the ingredients for the medium, adjustment to the formulation were made so that most of the nitrogen required for microbial growth came from bread and, most of all, protein hydrolysis was improved. The selected formulation contained 50% of bread and the incubation occurred with the enzymes E1 and E2, a complex mixture of both endo- and eso-proteases that highly efficiently degrade gluten in wheat dough (Rizzello et al., 2007), allowing the reduction of the supplements added to 10 g/l (Figure 2).

The protocol also comprised a step during which, before sterilization, the pH was normalized to 5.6 (Figure 2). This choice was made *i)* to ensure that the pH, which could change after the different incubations (time, temperature, enzyme used), was not a variable influencing microbial growth, and *ii)* because it is in the range of pH optimal for both yeasts and LAB growth (Gomes and Malcata, 1999; Kurtzman et al., 2011).

The performances of the optimized WBM were then tested on a wider range of microorganisms. The results from the LAB and yeasts enumeration on food matrices, reflected the behavior of the indicator strains, nevertheless the kinetics of growth of single LAB dissociates a little from *Lb. plantarum* LB1. The phenotypic properties of each strains have a significant impact on the performance of the strain therefore influencing growth parameters. Similar results among strains of the same species where indeed obtained during the formulation of a medium containing no animal-derived ingredients (Heenan et al., 2002). The reason behind the different adaptation of LAB strains to the medium (Figure 3) might be lying on the inadequate content of micronutrients. Phosphate, a basic component required to produce phospholipids of the microbial cell membranes and nucleic acids, and vitamins, especially water-soluble B-vitamins, are needed for optimum growth of LAB (Gomes and Malcata, 1999; Durso and Hutkins, 2003; Singh et al., 2017). As a matter of fact, when wasted bread was used as substrate for yeast biomass production, remarkable improvement where found with the supplementation with KH_2_PO_4_ and NH_4_SO_4_ (Benabda et al., 2018). Another possible explanation of the different behavior in WBM could be the origin of the strains. Indeed, all the strains isolated from faba bean (*P. pentosaceus* I76, I214, I02, I014, F01, *Pediococcus* sp. I56, *Leuc. mesenteroides* I57) (Coda et al., 2017) showed higher μ*max* compared to MRS, suggesting higher ability to adapt to sub-optimal conditions.

The WBM was designed to be a medium for a broader spectrum of microorganisms, therefore, the growth of species largely employed as starters in food industry was also assessed. The optimized WBM gave good results on the two species employed in yogurt production, however, since each microorganism has different requirements, changes in the formulation can be performed to meet the specific needs. For this reason, a different version of the medium, containing WPC instead of yeast extract (M_44_), was tested on *Lb. delbrueckii* subsp. *bulgaricus* and *St. thermophilus*. WPC is often used as main ingredient in media for dairy starters (Durso and Hutkins, 2003) and its use relies on the ability of those starters to utilize lactose and whey proteins as carbon and nitrogen sources (Gomes and Malcata, 1999; De Vos et al., 2011), which is why increased performances were reported.

Dairy industry starters also include fungi. Mold cultures are used for several types of cheeses, including all varieties of blue cheeses, or those with the white surface such as Brie and Camembert, but also in many Asian soy-based fermented foods as well as to produce European sausages and hams (Durso and Hutkins, 2003). Along with *Rhizopus*, *Aspergillus* and other *Penicillium* species, *P. roqueforti* is one of the starter cultures used in these processes and WBM resulted a suitable medium for its cultivation. Nevertheless, since food preservatives are part of the ingredients in industrial breads, their effect on fungal growth was also assessed. Propionic, sorbic and benzoic acids are among the most commonly used, and in accordance with our results, sorbic acid is more effective than propionic acid (Suhr and Nielsen, 2004). Even though inhibition was found at the highest concentrations (Figure 4), it could be considered irrelevant, since the growth exceeded that in PDA.

Finally, WBM was used to propagate wine starters with good results. The yeast *S. cerevisiae* Enol behaved like *S. cerevisiae* E10, and *O. oeni* O1, selected for malolactic fermentation of wine, showed similar performances in WBM and MRS. As a matter of fact, its growth is supported by a complex combination of amino acids, peptides, and fermentable carbohydrates, and even though most *O. oeni* strains require biotin, nicotine, thiamine, and pantothenic acid (De Vos et al., 2011), no limitation of growth was observed in WBM.

On a laboratory scale, considering enzymes and supplements, the production cost of WBM is approximately three times lower than that of the conventional media. WBM production collects the interest of the bakery, who needs to give added value to a waste, and the starter industry, who needs cheap substrates for growing microorganisms. Moreover, WBM could be directly produced by the industrial bakeries to provide microbial biomasses to be used for the production cycle. Since all the WBM ingredients are food grade, fresh cultures in WBM can be directly used for the inoculum, or alternatively, cells collected by centrifugation can be inoculated without washing.

Conclusively, the need for sustainable food productions led the scientific community to exploit more efficient ways of utilizing waste from food processing and WBM represents a realistic option for the valorization of bread waste, responding to the modern vision of circular economy.

## Data Availability Statement

The datasets generated for this study are available on request to the corresponding author.

## Author Contributions

AM conceived the idea. SC and LN first assessed the production of the medium. MV optimized the protocol and wrote the manuscript. CR designed the experiments and oversaw the writing process.

## Conflict of Interest

AM, SC, and LN are employed by Valle Fiorita Catering S.r.l. The remaining authors declare that the research was conducted in the absence of any commercial or financial relationships that could be construed as a potential conflict of interest.

## Abbreviations

CaP: calcium propionate
Ksor: potassium sorbate
LAB: lactic acid bacteria
MRS, de man: rogosa and sharpe
OD: optical density
OPA: *o*-phtaldialdehyde
WBM: wasted bread medium
WPC: whey protein concentrate
WPI: whey protein isolates.
